# Welcome to the Journal *HLRP: Health Literacy Research and Practice*

**DOI:** 10.3928/24748307-20170307-02

**Published:** 2017-03-23

**Authors:** Michael K. Paasche-Orlow, Cynthia Baur, Howard Cabral, The Editorial Board of HLRP: Health Literacy Research and Practice

Welcome to the journal *HLRP: Health Literacy Research and Practice*! Please join us in creating a forum for the dissemination of high-quality health literacy research and state-of-the art practices. Much work has been done to lay the foundations of this field, but we have only just begun to realize our potential impact. Health literacy has emerged as a dynamic discipline of inquiry and action—and working together, this journal can be a great catalyst to improve health.

This journal will be an interdisciplinary and international publication dedicated to promoting excellence in research and practice to advance the field of health literacy. Our aim is to attract a full range of investigators and practitioners engaged in health literacy research and practice, including those involved in a broad array of public health, health services, epidemiology, health communication, translational, educational, policy, interventional, implementation, and dissemination activities.

The journal has several goals. The first is to continue developing a robust field of health literacy research and practice. Given significant confusion around such fundamental concepts as definitions and measurement in the field of health literacy, theoretical work is still needed. Optimally, however, the journal will largely be empirically driven, a place to test our theories and opinions and share what can be learned. Achieving this goal entails unpacking complexity, avoiding the esoteric or the marginal, and questioning everything. This will not be questioning for the simple purpose of playing the devil's advocate. Rather, it will be a form of scientific skepticism that helps refine our thinking, germinates creative ideas, and promotes practical solutions.

A second goal of the journal is to promote investigations of how to improve people's lives by addressing health equity. The work of making all aspects of health, health care, and public health easier through empowerment, education, and system redesign is the work of social justice. In this journal, we will describe both the ways health literacy is a key causal mechanism of health disparities across the spectrum of social determinants of health and the efforts we undertake to use these insights to improve health for all.

A third goal of the journal is to encourage researchers who are early in their career, as well as practitioners, to share their views, experiences, and insights. Expanding our community by welcoming people who are new to publishing will help bring new ideas and energy to the field. Toward this end, we aim to create a review process that nurtures good ideas. This includes a separate channel for reviewing articles about dissemination and implementation of evidence-based and promising practices with reviewers who understand the complexity of implementation.

We hope you will join our effort to advance the field of health literacy. We seek empirical submissions from a full range of quantitative and qualitative methods in both a regular length and a shorter format. To stimulate improvements in practice, the journal will include Best Practice articles to share insights gained from implementation of health literacy policies, programs, or strategies to aid replication, dissemination, and sustainability. We also welcome your Review papers and Perspective pieces that capture your views on a full range of issues related to theory, methods, and policy. In addition, a submission type called Mission and Motivation provides a forum for an author's voice so that we may draw inspiration from each other, learn about the experiences that have attracted our colleagues to this field, and keep us all engaged. Please see the Information for Authors (www.healio.com/public-health/journals/hlrp/submit-an-article) for more information. We take an expansive view of the field of health literacy, but please email (hlrp@healio.com) if you are unsure about the relevance of your work to the scope of this journal.

We look forward to your submissions and thank you wholeheartedly in advance for your submissions and for any assistance you may be able to give us in reviewing manuscripts. The Editors will be rating all reviews and will make charitable donations to adult literacy programs in honor of our highest rated reviewers!

This is a mission-driven endeavor. Please let us know all ideas you may have to make this a successful venture.

